# Knowledge of and access to frontline workers among poor, rural households in Amhara region, Ethiopia: a mixed-methods study

**DOI:** 10.1186/s12889-022-14594-8

**Published:** 2022-11-25

**Authors:** Sarah Quinones, Tia Palermo, Maja Gavrilovic, Vincenzo Vinci, Frank Otchere, Essa Chanie Mussa, the ISNP Evaluation Team, Gustavo Angeles, Elsa Valli, Jennifer Waidler, Getinet Tadele, Sewareg Adamu, Teketel Abebe, Yenenesh Tadesse, Feredu Nega, Mesay Kebede, Fekadu Muluye, Alene Matsentu, Daniel Aklilu, Ana Gabriela Guerrero Serdan, Lisa-Marie Ouedraogo, Getachew Berhanu Kebede

**Affiliations:** 1 Department of Epidemiology and Environmental Health, University at Buffalo (State University of New York), 270 Farber Hall, Buffalo, NY 14214-8001 USA; 2 Via Degli Alfani 58, 50121 Florence, Italy; 3 Maastricht Graduate School of Governance, Maastricht University/UNU-Merit, Maastricht, Netherlands; 4 UNICEF, Ho Chi Minh City, Vietnam; 5 Department of Agricultural Economics, University of Gondar, Gondar, Ethiopia

**Keywords:** PSNP, HEW, Access, Community social workers, Health, Social services, ISNP

## Abstract

**Background:**

Social protection programmes have effectively reduced poverty and improved food security. However, the effects of poverty require an intersectoral approach to adequately address poor nutrition and health. Identifying gaps in knowledge and access to frontline workers who oversee these integrations is critical for understanding the potential for integrated social protection programming to improve these outcomes. We measured levels of social protection programme participants’ knowledge of and interaction with social workers (SWs) and health extension workers (HEWs) in rural Ethiopia.

**Methods:**

This mixed-methods study uses cross-sectional data from the baseline survey of a quasi-experimental impact evaluation among a sample of 5,036 households participating in Ethiopia’s Productive Safety Net Programme. Qualitative interviews include key informant interviews, in depth interviews and focus group discussions with caregivers, community members, frontline agents, and stakeholders. Using data from household questionnaires administered to household heads, quantitative analyses include univariate and bivariate descriptive statistics as well as mutually-adjusted multivariable logistic regression analyses to estimate adjusted odds ratios and 95% confidence intervals for household sociodemographic characteristics associated with 1) knowledge of SWs and HEWs and 2) interaction with SWs and HEWs in their communities. Qualitative data were analysed using thematic analysis combining both a fluid and more structured coding processes to unpack the important topics within the data supported by illustrative quotes.

**Results:**

Our results show that knowledge of and interaction with SWs is limited while many knew of and interacted with HEWs quite regularly. Interactions with SWs were negatively associated with increased household size and living in Dewa Chefa. Factors associated with increased knowledge of and interaction with HEWs include having children under the age of 5 years in the household, having health insurance, and having a formal education. Qualitative analyses suggest that SWs are limited by overwhelming caseloads, limited resources to carry out their work, and high staff turnover. However, SWs are considered highly valuable in the communities where they work.

**Conclusions:**

While most of the participants reported knowing their HEW, there is room for improvement, especially around household engagement with HEWs. Although SWs support the ISNP in the treatment districts only and not formally incorporated into the structure in the region, our findings highlight a need to provide greater support to SWs to effectively facilitate improvements in health and nutritional outcomes among vulnerable households.

**Trial registration:**

Pan African Clinical Trial Registry (PACTR201902876946874) and the Registry for International Development Impact Evaluations (RIDIE-STUDY-ID-5bf27eb0404a0).

**Supplementary Information:**

The online version contains supplementary material available at 10.1186/s12889-022-14594-8.

## Background

The Government of Ethiopia (GoE) successfully reduced the country’s poverty rate from 30% in 2011 to 24% in 2016, yet 30 million Ethiopians remain in poverty, especially in rural regions [1]. Multidimensional poverty incidence was 36.8% and 91.8% in urban and rural households of Ethiopia in 2016, respectively [2]. Health-related challenges also exist. For example, pregnancy-related mortality ratio was 412 per 100,000 live births and the infant mortality rate was 48 per 1,000 live births in 2016 [3]. Levels of child malnutrition vary considerably across regions with 46% of children under the age of 5 years in Amhara stunted compared to 15% in Addis Ababa. The percentage of women exclusively breastfeeding for the first 6 months of their child’s life has improved from 52% in 2011 to 58% in 2016 [3], but this still falls short of exclusive breastfeeding recommendations in the first six months of life. Despite improvements in health outcomes and reductions in poverty across Ethiopia, many challenges remain and vulnerability of rural populations still presents a concern. Thus, examination of approaches to alleviate poverty and its accompanying vulnerabilities, particularly in rural Ethiopia, is warranted.

### Social protection in Ethiopia

One major strategy to achieve the Sustainable Development Goals (SDGs) related to poverty and health is social protection programming [4]. The Productive Safety Net Programme (PSNP), started in 2005, is Ethiopia’s flagship social protection programme. The PSNP aims to address determinants of poverty to promote the livelihoods of Ethiopia’s most vulnerable, extremely poor, and food insecure households [5]. The PSNP has reached more than 8 million people since its inception [1, 6] and has traditionally comprised two groups: those eligible for Direct Support (permanent [PDS]) and Public Works (PW) groups. The latter group participates in PW programmes to maintain eligibility for cash payments, while the former comprises households with no able-bodied members and thus receives unconditional cash transfers. A third group was introduced under the fourth phase of the PSNP (PSNP4; 2015–2020): the temporary direct support (TDS) group. This group includes pregnant and lactating women from PW households and caregivers of malnourished children. Pregnant and lactating women are exempted from work requirements from the time the pregnancy is reported until the child is 12 months of age, provided that the child does not have malnutrition problems. On the other hand, caretakers in PW households with malnourished children transition to TDS as soon as a malnourished child is identified until the child fully recovers from acute malnutrition.

Under integrated social protection, complementary programmes across sectors are meant to help households leverage the cash payments from the PSNP to improve livelihoods, health, and nutrition. Integrated social protection programming is increasingly implemented to more holistically address multidimensional poverty and improve well-being. However, the success of these intersectoral endeavors depends on key cadres implementing those linkages. In Ethiopia, these frontline workers include Social Workers (SWs), Health Extension Workers (HEWs), and Development Agents (DAs).

An example of an integrated social protection pilot aiming to strengthen intersectoral linkages is the Integrated Safety Net Programme (ISNP) being piloted between 2019 and 2023 within selected PSNP districts (locally referred to as “woredas”) in the Amhara region. Implemented by the Ministry of Labor and Social Affairs (MoLSA) and the Bureau of Labor and Social Affairs (BoLSA) with technical assistance from UNICEF, the ISNP targets PSNP households which already receive cash transfers with additional linkages of services around nutrition and health and seeks to facilitate enrolment into community-based health insurance (CBHI). An innovative feature of the PSNP allocates certain co-responsibilities related to basic health, nutrition, and education services to TDS clients. Programme aims are achieved through collaborations between frontline workers (SWs, HEWs, and DAs) and woreda offices to promote access to and utilisation of these essential services among programme beneficiaries. The specific components of the ISNP include: 1) Behaviour Change Communication (BCC) sessions; 2) facilitation of enrolment into the CBHI among PW households and exempting PDS clients from paying the enrolment premium; 3) Case management by SWs to support linkages between PSNP clients and health and social services, informing clients of their co-responsibilities (including children’s school enrolment and attendance and other health related service visits), monitoring compliance with co-responsibilities and providing follow-up advice or support in cases of non-compliance; and 4) a Management Information System (MIS) intended to allow client information and needs to be stored and shared more efficiently across programmes. The ISNP seeks to enhance the collaboration among frontline workers to improve engagement, introduce strengthened messaging, and provide and facilitate BCC sessions for improved knowledge of health and nutrition services and needs. HEWs and SWs serve critical roles in the programme’s delivery. In the ISNP, frontline agents, specifically SWs, are tasked with the implementation of inter-sectoral collaboration and linkages between beneficiaries and social services. These workers represent pivotal intermediaries between PSNP households and programme components that operate at many levels. Thus, their understanding of the programme, its objectives, their roles and services rendered, and knowledge of the populations they serve are critical to the successful implementation of the programmes that they operate under. These cadres must not only be well resourced and trained, but program participants must be aware of their existence and the types of help they can offer.

Findings from a previous integrated social protection pilot, Ethiopia’s Improved Nutrition through Integrated Basic Social Services and Social Cash Transfer (IN-SCT) pilot programme, suggest that frontline workers were aware of their roles, the collaborations needed to promote intersectoral linkages, and could readily identify barriers and facilitators to the successful implementation of the IN-SCT programme [7]. However, actual capacity to carry out their work was hampered by technical restraints, high staff turnover, heavy workloads, and low pay. Similarly, high turnover of DAs, HEWs, school principals, and others was identified as a barrier to effective SW service delivery by an evaluation of the Tigray Social Cash Transfer Pilot programme (SCTPP), a social protection programme that sought to improve access to basic social services among clients [8].

### Frontline workers

HEWs in Ethiopia considered themselves both competent and reliable although many received on the job training and even more received inadequate pre-service training [9]. HEWs considered monitoring data and acting as clinical preceptors to be critical to service delivery and improved outcomes but also that these tasks were performed too infrequently [9]. DAs in the Southern Nations, Nationalities, and Peoples (SSNP) region of Ethiopia reported overwhelming workloads, the need to supplement income with additional jobs which compounds the encumbering nature of their work, and experienced high turnover rates as a result of these factors [10].

Knowledge and attitudes among the populations served by SWs and HEWs can also present demand-side barriers to effective service delivery and uptake. A study in Ethiopia assessed client knowledge of and interaction with their community DAs, SW, and HEWs in Oromia and SNNP regions and found that clients in SNNP were twice as likely to know their HEW than their Oromia counterparts (83% vs. 41%). Knowledge of SWs in these regions was significantly lower as 8% and 11% of clients reported knowing their SWs in Oromia and SNNP, respectively [7]. A cross-sectional community-based study among households in the district of Abuna Gindberet (Oromia region) found that less than half of the respondents had knowledge of the health extension services, and even fewer (39%) utilised these services. Lack of knowledge was associated with a 75% reduced odds of service utilization when compared to those who were considered knowledgeable [11]. Findings from other studies further underscore the positive relationship between increased education and information on health services and subsequent utilisation [12, 13]. Transportation, distance, opportunity costs, and cultural norms were all found to be barriers to health seeking behaviour among individuals in low- and middle- income countries [12]. Taken together, these reported barriers suggest that a comprehensive, integrated approach is needed to address the myriad barriers to health care access and utilisation.

Direct and indirect challenges to the daily operations and overall tasks of frontline workers described above highlight the importance of considering both demand- and supply-side barriers to access and utilisation of social services. In the current paper, we aim to 1) assess baseline rates of PSNP beneficiary knowledge of and interactions with SWs and HEWs; 2) examine the associations between sociodemographic factors, knowledge of and interactions with these frontline workers; and 3) assess challenges faced by frontline workers using a mixed-method approach.

### Conceptual framework

This research is informed by the conceptual framework illustrated in Supplementary Fig. 1. This conceptual framework is intended to provide a general guide as to how intersectoral linkages to social protection programmes work at various levels and has been adapted from the work of Vinci and Roelen [14]. Given the complex intersectoral and multi-level operations of the ISNP and other social protection programmes, it is imperative to understand, at all levels, how knowledge of and interactions with frontline agents may be determined by programme objectives, implementation, and effectiveness. At the national level, establishment of intersectoral linkages is largely influenced by political economy factors, government and administrative structures, and capacity of institutions. Establishment of linkages can occur as a result of policy windows which in some instances are informed by robust evidence or when are imposed by national strategies. Memoranda of understanding between ministries is one way to increase the likelihood of success of integrated, intersectoral initiatives. To further ensure the success of these linkages, social protection and the linked programmes must be adequately financed in the national budget and, subsequently, these allocations must be disbursed to sub-national levels and implementing agencies in a timely fashion. Moreover, specific bodies responsible for the facilitation of cross-sectoral programming need to be adequately funded and with adequate institutional capacity.

At the regional level, programme personnel must be adequate and its roles and responsibilities clear. This contributes to promote intersectoral planning, delivery, and monitoring of social protection interventions. Intersectoral work would benefit from coordination mechanisms such as operational and programmatic guidance for staff and tools for joint work planning. Timely disbursement of programme funding and clear communication of programme objectives to district-level staff can also ensure proper programme implementation and successful linkages across sectors. Moreover, monitoring and evaluation mechanisms can contribute to inform programme outcomes and activities about what is working well and what is not, so that adjustments can be made accordingly.

At the community level, capacity of government workers to perform their respective activities is influenced by a clarity of roles and responsibilities, coordination mechanisms for horizontal intersectoral work, and improved awareness of policy and programmes objectives. That is to say, the cadres responsible for the facilitation of cross-sectoral programming need to be adequately funded, have sufficient space in their daily activities to carry out programmatic linkages in a quality manner, and must receive adequate communication and information about target populations, programme objectives and motivation for linked programming, so that such programming can be carried out as intended. Similarly, performance and motivations of frontline workers could be augmented with incentives for intersectoral collaboration, improved training, reduced overlap of frontline worker responsibilities, better understanding of roles and responsibilities, and transportation met with the ability to meet target populations.

Intersectoral collaboration is influenced (moderated) by numerous operational factors including quality of institutions (from the separate, linked sectors) involved in integrated programme implementation and quality of the implementation itself. Functioning community structures contribute to quality of services provided as well as quality of programme implementation. In order to ensure this component, services should be timely, relevant, and of acceptable quality, grievance mechanisms should be clarified to clients, and informal support systems and local political administration function should be recognized and integrated into procedures accordingly. Infrastructure is an essential component to implementation and institutional quality. For example, distance to services can influence client perceptions and attitudes and affect frontline worker ability to engage with the most vulnerable households and monitor co-responsibilities.

## Methods

### Study design and setting

Data for this cross-sectional study come from the first round of data collection (December 2018 to February 2019) for a quasi-experimental, mixed-methods impact evaluation of the ISNP pilot in the Amhara Region of Ethiopia [15] being carried out by the UNICEF Office of Research (OoR) Innocenti, University at Buffalo, and Frontieri (formerly known as BDS Centre for Development Research), in collaboration with UNICEF Ethiopia (ECO).

The study was conducted in four purposively selected *woredas* (districts): Libo Kemkem, Dewa Chefa, Ebinat, and Artuma Fursi.

### Sample and procedures

**Fig. 1:**
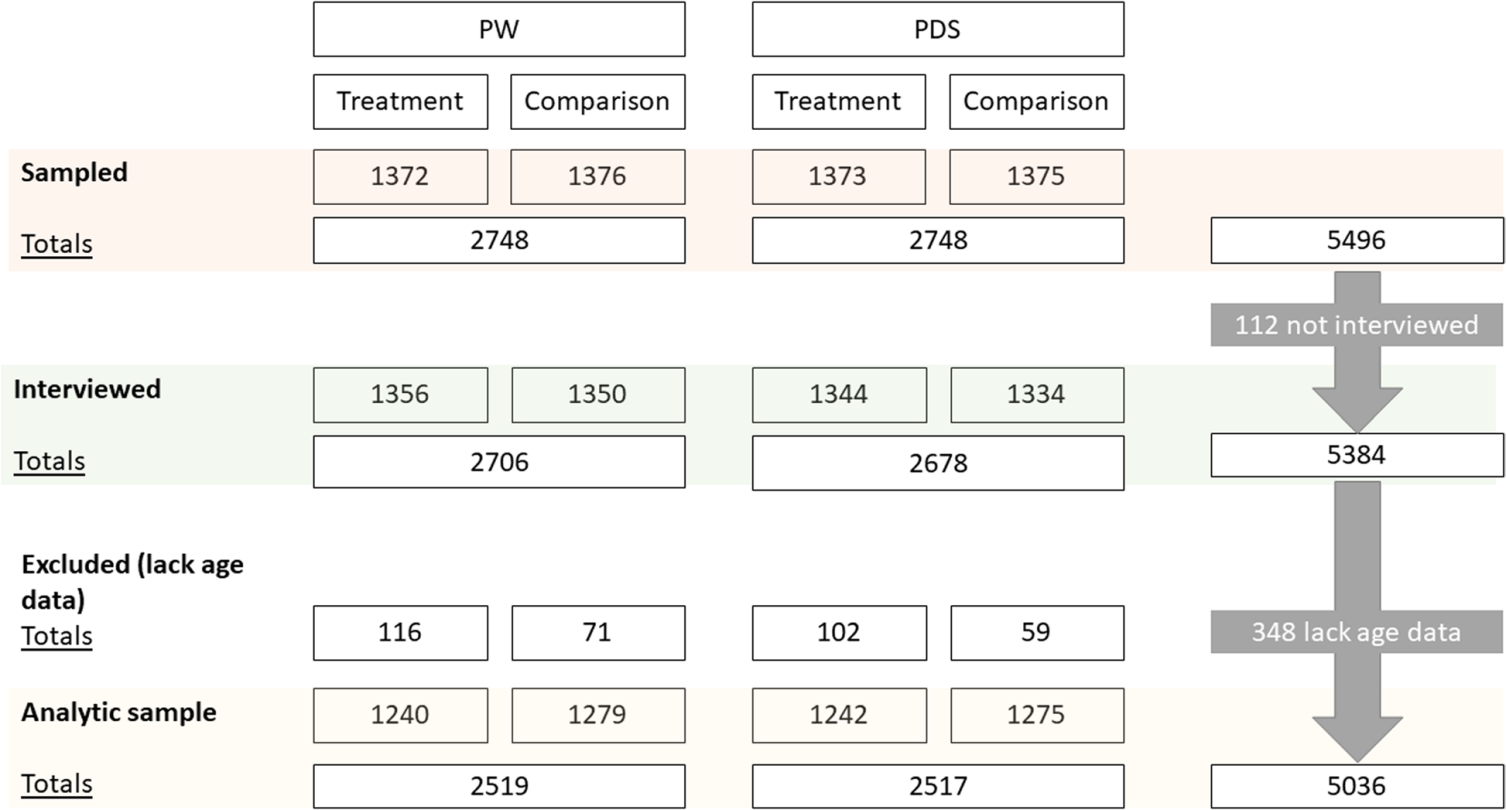
CONSORT diagram for study sample in Amhara, Ethiopia

Eligibility criteria for inclusion in the study include 1) households that participate in the PSNP; 2) reside in woredas selected for the study; and 3) have complete information on all relevant sociodemographic variables explored in this study. Households were excluded if they did not meet these criteria (see exclusions in Fig. 1). While the current study is observational, it draws on baseline data from an impact evaluation, where sample size was determined based on expected impacts on key indicators, including use of health services, enrolment in CBHI, vaccination rates, and receipt of antenatal care. Based on power calculations, it was determined that a minimum of 5,400 households should be sampled with at least 2,700 in each of the treatment and comparison arms, and the sample was further stratified and split evenly between PW and PDS households in each arm [16]. To account for some households that may need replacement due to inability to locate them or inaccurate administrative documents, we selected a total of 5,496 households as targets for sampling. The sampling frame included all PSNP households included in PSNP administrative data in each study woreda as of October 2018. Simple random sampling was used to select the number of allocated households at the *kebele* level from each category of households. Sample selection for this study is shown in Fig. 1. Of the targeted households, 5,384 were successfully interviewed. More detailed information on the study design and sampling frame of the evaluation is available in the Supplementary Table 1.

Quantitative data were collected via household surveys administered to the main female caregiver in the household or the most knowledgeable person in the household if the main female caregiver was not available.

To further understand the performance of key frontline workers and the relationships of interest, the same cohort of households was included to participate in qualitative interviews at three different points (baseline, 13-month and 24-month waves). Qualitative data were collected in two treatment *woredas* —one *kebele* in each *woreda* (Shemo *kebele* in Libo Kemkem and Gula *kebele* in Dewa Chefa). These research sites were purposively selected because of their relatively high investment in the social workforce, prevalence of CBHI enrolment, and good accessibility to research teams. Qualitative methods comprised in-depth interviews (IDIs) with PSNP clients (32 total), key informant interviews (KIIs) with government staff and frontline workers (18 total), and focus groups discussions (FGDs) with community care coalitions (CCC; 2 total). A stratified sample for IDIs included discussions with female caregivers in the following three categories: 1) PW and PDS beneficiary households with at least one child under age 18 years (12 total); 2) PW households with pregnant and lactating women (12 total); and 3) caregivers in households with malnourished children aged 6–59 months (6 total). Within each category, eligible study participants were randomly selected from a subsample of the larger quantitative evaluation sample.

Socio-economic characteristics of the study participants varied depending on the PSNP client status. While PDS clients were generally older and without young children (average age of 70 years), the PW clients mainly comprised adults aged 40 years on average, and all had children (four on average per family). On average, clients were involved in the programme for four years. IDIs with PSNP beneficiary households in the treatment woredas focused on beneficiaries’ experiences, perceptions and awareness of the pilot programme’s operational features, such as targeting processes, payment delivery, case management and referral systems, rights and grievance mechanisms, assess access to social services (including health insurance) and interaction with frontline workers. KIIs were undertaken with programme staff at *regional* (e.g., BoLSA Officer and Bureau of Health Officer), and *woreda* level (e.g., WoLSA Officer, ISNP, CBHI and PSNP coordinators). At the *kebele* level, interviews were conducted with HEWs, SWs and DAs to discuss perceptions on ISNP implementation processes and procedures, including success factors and challenges. Finally, FGDs were conducted with CCCs at the *kebele* level to examine their roles and experiences in service delivery and cross-sectoral coordination. Interviews were conducted in Amharic and Oromifa. A coding framework was developed to guide a thematic analysis of data to identify patterns and main themes in key areas of interest. A code book was developed using a priori themes from the interview guides (20 themes in total) and supplemented with sub-themes that emerged during data analysis (81 sub-themes in total).

### Ethics statement

This study was approved by the Amhara Public Health Institute Research Ethics Review Committee (Ref # 03/192/2018). Informed consent was obtained verbally from all respondents aged 18 years and above, and caregiver or parental consent and youth assent was obtained verbally for all youth aged 12–17 years. Outcome of the consent process was then entered into electronic tablets by survey enumerators.

### Measures

Primary outcomes of interest include knowledge of and interactions with frontline agents. Respondents were asked if they know their SW, HEW, or DA. If they had knowledge of these frontline workers, the beneficiaries were then asked what, if any, contact they ever had with a given agent and, further, whether the contact occurred in the past three months (what we term ‘recent’). Contact may have occurred in the home, in the community, or at a health post. An affirmative response to any of the questions was coded as a “1” whereas negative responses were coded “0.” In the case that respondents reported having no knowledge of these frontline agents in their communities, they were excluded from further questions on interactions, visits, and contacts. Additionally, questions regarding food demonstration attendance and visits to a health post were asked to assess utilisation of services among the entire sample of PSNP clients. Household- and individual-level explanatory variables in these analyses include: female household head, children under the age of 5 years in the household, enrolment in CBHI, education, household size, respondent sex and age, an asset index, distressed asset sale, number of children 24–35 months old in the household, and district of residence. The asset index was created with information on housing materials, electricity, transportation, cooking instruments, and agricultural tools using principal components analysis, similar to the approach of Filmer and Pritchett [17]. Distressed asset sale (DAS) is a negative coping strategy defined as the sale of critical or valuable household resources because of a shock to the household [18–20]. DAS was defined by asking if households had, in the past two years, been forced to sell any productive assets or consumer durables or rent out or exchange any land or livestock in order to meet needs related to food or cash for emergency needs such as health care. An affirmative response to any of these questions was coded as “1” and negative responses were coded to “0.” Row totals were then calculated across all responses for each participant to generate a cumulative score of DAS experiences (range 0–8). We then created a categorical variable for DAS if the respondent indicated yes to two or more items on this scale. Female household head, children under 5 years old in the household, enrolment in CBHI, having any education, respondent sex, and district of residence were operationalized as dichotomous variables while the remaining explanatory variables (household size, respondent age, and number of children 24–35 months old) were continuous.

### Statistical analyses

We used a complete-case approach for our analytical sample. We examined background characteristics and outcomes among the pooled sample of treatment (CBHI + PSNP) and comparison (PSNP only) groups, since these are baseline data collected prior to ISNP programme implementation. Because of the stratified sampling approach for PDS and PW samples, we analyze these groups separately. We first ran descriptive analyses to summarise sociodemographic characteristics and outcomes of interest. Next, we examined the relationships between the sociodemographic characteristics with knowledge of and access to these frontline actors and services using mutually-adjusted multivariate logistic regression models that estimate adjusted odds ratios (aOR) and 95% confidence intervals (CI). We considered results to be statistically significant at an alpha level of 0.05. Model fitness was assessed using the Hosmer–Lemeshow goodness-of-fit test [21] and multicollinearity was examined using the variance inflation factor (VIF) of all explanatory variables.

A robustness check was employed to evaluate the effect stability of the explanatory variables on the outcomes [22]. Among this population, it is common for an individual to lack knowledge of their exact age when asked in an interview. Resulting from this uncertainty of age, approximately 7% of the sample lacked complete data. Results from the sub-sample with no missing age information was compared to the results from the full sample with 7% missing age information. Based on the results of the robustness check and subsequent comparisons of means and regressions the results with non-missing age data was presented. More details on the results of the robustness checks are presented in Supplementary Table 2. All quantitative analyses were conducted using Stata version 16 [23].

### Qualitative analysis

For qualitative analysis, quotes and responses from KIIs, FGDs, and IDIs are considered and summarised relative to the outcomes included in these baseline analyses. Thematic analysis was used to analyse qualitative data in two phases: 1) rapid initial analysis to document observations during fieldwork and 2) in-depth analysis of transcripts to increase overall understanding of the programme delivery and its influence on participants’ lives. During the in-depth phase, the qualitative research team developed also analytic summaries for all participants. These summaries were organised around key themes and evaluation questions, to simultaneously code the data to identify patterns in the key areas of interest to the programme while also tracking changes and narratives over time. Transcripts and analytical summaries were coded and analysed using Atlas.ti software. A codebook was created using a priori themes from the interview guides and supplemented with themes that emerged during two phases of data analysis. Finally, illustrative quotes in the transcripts were selected to reflect key themes and provide evidence of results.

### Availability of data and materials

Data are not currently publicly available and cannot be made available upon request due to Government processes (the Government of Ethiopia and UNICEF jointly own the data). Data are expected to be made publicly available, subject to approval by the Government of Ethiopia and UNICEF, no sooner than one year after the publication of the final impact evaluation report.

All methods were carried out in accordance with relevant guidelines and regulations.

## Results

Out of 5,384 households surveyed, a total of 5,036 households with complete data on measures of interest were included in the sub-sample for analysis (see Fig. 1 and Table 1). Based on a VIF threshold of 2, we found no multicollinearity in our models and goodness-of-fit was demonstrated by the Hosmer–Lemeshow test. This sample was comprised of observations from 218 male (4%) and 4,818 female caregivers (96%). On average, PW households had larger households (mean = 5) than PDS households (mean = 3). Most (66%) of the PDS households were female-headed compared to less than half of the PW households (36%). The average age of household respondents was approximately 46 years old with PDS respondents generally older than PW respondents. Most (93%) reported having no high school education. Approximately one in five respondents reported having any DAS.

**Table 1:** Sociodemographic characteristics of participant households pooled and by participant status (*N* = 5,036)

		% or mean ± SE		
		PW	PDS	Pooled
	Household size	5 ± 0.10	3.02 ± 0.08	4.44 ± 0.11
	Has child(ren) under 5 years	41	17	34
	Covered by CBHI	70	50	65
	Household head is female	36	66	44
	Has had any distress asset sale	21	20	21
	Asset index	-0.14 ± 0.07	-0.39 ± 0.07	-0.21 ± 0.05
	No high school education	92	93	93
	Less than primary school	6	4	6
	Respondent is female	97	94	96
	Age of respondent	42.1 ± 0.32	55.6 ± 0.52	45.9 ± 0.54
District	Libo Kemkem	17	18	17
	Ebinat	26	34	28
	Dewa Chefa	22	18	21
	Artuma Fursi	35	30	34
	N	2,519	2,517	5,036

*CBHI* Community-based health insurances, *PDS* Public Direct Support, *PW* Public Works, *SE* Linearized standard error.

Proportions of participants’ reported knowledge of and interactions with the various frontline agents and service utilization are presented in Table 2 by beneficiary type.

Most (92%) of these PSNP households do not know the SW working in their areas (Table 2). Of these respondents who reported knowing their SW, 40% had any contacts with the SW. Compared to 13% of PW households, 18% of PDS households reported being visited by a SW in the past 3 months. Further, 4% of PW households attended a food demonstration (FD) compared to 1% of PDS households (Table 2).

**Table 2:** Proportions of participant knowledge of and interactions with health extension workers and social workers, pooled and by participant status (*N* = 5,035)

	PW	PDS	Pooled
Knows HEW	0.62	0.45	0.57
Contacted by HEW (past 3 months)	0.38	0.36	0.38
Ever visited by HEW	0.43	0.47	0.44
Visited by HEW in past 3 months	0.24	0.27	0.24
Contacted by HEW outside of home	0.20	0.19	0.20
Ever visited HP	0.43	0.32	0.40
Visited HP in past 3 months	0.11	0.07	0.10
Received health advice at HP	0.05	0.03	0.04
Knows HDA	0.11	0.08	0.10
Contacted by HDA	0.37	0.37	0.37
Knows SW	0.08	0.07	0.08
Had any contact with SW	0.40	0.40	0.40
Ever visited by SW	0.19	0.31	0.22
Visited by SW in past 3 months	0.13	0.18	0.14
Contacted by SW outside of home	0.19	0.17	0.18
Attended FD	0.04	0.01	0.03
Attended FD in past 3 months	0.01	0.00	0.01
*N*	2,519	2,516	5,035

*HEW* Health Extension Worker, *SW* Social Worker, *FD* Food Demonstration, *HDA* Head Development Agent, *HP* Health Post

The IDIs show that SWs perform a broad range of services and tasks related to the integrated service provision.

“The SW facilitate the PSNP payment and she mobilizes us to participate in PW.” (IDI with public works beneficiary, Libo Kemkem)

*“We ask her to make the payment as quickly as possible when we face financial difficulties. She knows when we have problems. Even when I am supposed to collect the payment in a month or two months time, she prepares and comes during the market days and informs me about the timing of the payment. This is because she is concerned if I miss the payment. There are improvements”. (IDI with permanent direct support beneficiary, Dewa Chefa)*

Interviews with programme personnel and PSNP clients who know of and have interacted with the SW in their community, value the work and support SWs provide to the beneficiaries and community as a whole:

“The social workers are very good in covering different problems in the society. They are very responsible in delivering support on time. They are very committed.” (KII with BoWCA staff, Dewa Chefa)

“Nobody supports us except the social worker”. (IDI with pregnant woman, Libo Kemkem)

“Yes, social worker counsels and supports me to improve my health and to save money and to be able to feed ourselves like anybody else.” (IDI with caregiver with malnourished child, Libo Kemkem)

SWs face many challenges in their work, including very high workloads; insufficient material support to implement their tasks, particularly home visits; and limited training. The sometimes temporary contracts in terms of employment status and major delays in salary payment have been reported to affect their level of commitment, motivation and work performance.

“They face material problems, like umbrella and bags to do their home-to-home activities as desired.” (KII with CBHI Coordinator, Dewa Chefa)

“There are social workers who are hired temporarily. Their achievements would improve if they are hired permanently and have better capacity.” (KII with PSNP Coordinator, Libo Kemkem)

“Now they are paid 1500 birr. But this is not sufficient. Even if the salary is too small it arrives lately. Sometimes this makes them discouraged.” (KII with BoWCA staff, Libo Kemkem)

Approximately 57% of household survey respondents reported knowing their community HEW (Table 2). Among those that know their HEW, 44% had ever been visited by the HEW and 38% reported having any contact with the HEW in the 3 months prior to the interview. Less than half (40%) of all caregivers ever visited a health post and 10% visited in the past three months. Most participants (96%) had not received any advice related to breastfeeding, child feeding, or nutrition during their visit. No males in this sample reported receiving health advice in either beneficiary category [data not shown]. Overall, PW households knew and interacted with frontline agents more so than PDS households (Table 2).

The qualitative research found that HEWs provide a broad menu of support and services to the community.

*“They give advice to implement family planning, to visit the health center every month if we are pregnant, how to treat our child, how to use contraceptive, about health checkup and the like”. (IDI with pregnant woman, Libo Kemkem)*

“They provide treatments and supports for our children. They usually give children vitamins, supplementary foods, biscuits and syrups.” (IDI with public works beneficiary, Dewa Chefa)

Within the PSNP, HEWs also play an important role in programme administration and targeting by transitioning pregnant and lactating women and cargivers of malnourished children from PW into TDS, as well as screening PSNP clients for CBHI membership and fee waivers.

“Without the report from the health extension worker, the agriculture sector will not be able to give leave to the pregnant women from public works.” (KII with Social Workers, Dewa Chefa)

“It is us who are doing screening of beneficiaries. We and kebele leaders work to enroll the community in the CBHI.” (KII with Health Extension Worker, Libo Kemkem)

Clients that have had interactions with HEWs view their role in a positive light. PSNP beneficiaries also reported improvements in their awareness and knowledge of key health issues, resulting in positive changes in behavior related to nutrition and health because of their interactions with HEWs:

*“For instance, so far I used to throw wastes just outside, but now, after they taught me I collect the waste and burn it. In addition, we clean what we eat on and what we drink with.” (IDI with pregnant woman, Libo Kemkem)*

“HEWs changed my views about latrine, child feeding practice and ANC service.” (IDI with public works beneficiary, Libo Kemkem)

*“HEWs support helped me. I got contraceptive services when I was newly married and this gave me opportunity to time and space births. My children become healthy because the HEW taught us how to feed and keep personal hygiene.” (IDI with public works beneficiary, Dewa Chefa)*

Despite these positive examples, both the PSNP clients and frontline workers, highlighted several challenges to adequate service provision.

*“Because they are so busy. One health extension worker has engaged in 12 work processes. There is no one who is as busy like health extension workers. There is vaccination, family planning, health package, development group, nutrition that health extension workers are engaged with. They have so many commitments*.” *(KII with CBHI Coordinator, Libo Kemkem)*

HEWs were expected to spend an allotted percentage of their time at the health posts, providing direct medical services to the community, however, these expectations were not always fulfilled:

*“The problem with the support that we receive from HEWs is they do not live in our community. When a woman wants family planning they will not be present in their work place all the time. We face problem to get service on time. The health extension workers don’t treat sick children. They refer them to health center. They should stay at health post.” (IDI with caregiver with malnourished child, Libo Kemkem)*

Ten percent of households reported knowing the HDA leader, and 37% were contacted by an HDA. HDAs could not properly facilitate the BCC sessions as they lacked clear understanding of the sessions’ purposes although they were rather successful in transitioning pregnant and lactating women into TDS.

“As I told you earlier the training in BCC itself is not clear to me. But to tell you the truth we didn’t provide any BCC sessions to our client.” (KII with Development Agent, Dewa Chefa)

### Characteristics associated with access to health extension workers

Table 3 presents the results of the mutually-adjusted logistic regression models for access to health workers and services among PDS beneficiaries. Client household characteristics positively associated with knowing the HEW include household size, having children under 5 years of age in the household, CBHI coverage, having any DAS in the past 2 years, and higher asset index. Residents in Artuma Fursi were less likely to know HEWs than residents of Libo Kemkem (p < 0.05). Factors positively associated with recent contact with an HEW are household size and increasing asset index. Residing in Dewa Chefa or Artuma Fursi (versus Libo Kemkem) and having a higher number of children 24–35 months old were negatively associated with recent HEW contact. Having residence in Ebinat relative to Libo Kemkem was shown to be negatively associated with visits by an HEW in the past 3 months, while increasing household size was positively associated with this outcome. Characteristics positively associated with having any contact with an HEW outside of the home are asset index and having any education relative to no formal education. Residing in either Dewa Chefa or Artuma Fursi (versus Libo Kemkem) was negatively associated with having contact with an HEW outside the home. Increased household size, children under 5 years living in the household, CBHI coverage, any DAS, and higher asset index are positively associated with ever visiting a health post. Characteristics positively associated with recent visitation to a health post include having children under the age of 5 years in the household, CBHI coverage, having any versus no education, and increased asset index. Living in Ebinat or Artuma Fursi relative to Libo Kemkem was negatively associated with visiting a health post in the past 3 months. Increasing household size, having children under the age of 5 in the household and having any versus no formal education are positively associated with receiving health advice at the health post. Characteristics positively associated with knowledge of the HDA include any DAS, increased asset index, having any education, and residing in Dewa Chefa versus Libo Kemkem. Individuals residing in Ebinat were less likely to know the HDA than those residing in Libo Kemkem.

**Table 3:** Adjusted odds ratios and 95% confidence intervals for the associations between household characteristics and knowledge of and interaction with health extension workers for PDS households (*N* = 2,493)

	**Knows HEW**	**Any contact with HEW (past 3 months)**	**Ever visited by HEW**	**Visited by HEW (past 3 months)**	**Contact with HEW outside home**	**Visited HP**	**Visited HP (past 3 months)**	**Received health advice at HP**	**Knows HDA**	**Had contact with HDA**
Household size	1.15	1.12	1.05	1.14	1.09	1.08	1.05	1.17	1.07	1.04
	[1.07—1.24]^**^	[1.03—1.23]^*^	[0.98—1.13]	[1.05—1.24]^**^	[0.99—1.21]	[1.01—1.16]*	[0.95—1.16]	[1.03—1.32]^*^	[0.98—1.15]	[0.84—1.27]
Children under 5 in the household	1.97	1.24	1.07	1.06	1.23	1.63	2.08	4.26	1.12	1.05
	[1.33—2.92]**	[0.86—1.78]	[0.68—1.68]	[0.69—1.64]	[0.88—1.71]	[1.20—2.20] ^**^	[1.35—3.21] ^**^	[1.71—10.62]^**^	[0.71—1.75]	[0.54—2.02]
Covered by CBHI	1.71	0.95	1.05	0.90	0.95	1.48	1.88	1.40	1.31	1.19
	[1.40—2.09]**	[0.74—1.22]	[0.77—1.44]	[0.67—1.21]	[0.66—1.39]	[1.22—1.79] ^**^	[1.27—2.77] ^**^	[0.67—2.94]	[0.90—1.89]	[0.53—2.65]
Head is female	1.25	1.20	0.97	1.27	1.10	0.93	0.65	0.82	1.10	0.64
	[0.97—1.62]	[0.85—1.70]	[0.72—1.30]	[0.89—1.82]	[0.71—1.71]	[0.73—1.19]	[0.45—0.94]*	[0.41—1.66]	[0.71—1.69]	[0.35—1.16]
Any distress asset sale (DAS)	1.40	0.87	1.04	0.99	1.09	1.57	1.36	0.88	2.16	1.20
	[1.05—1.87]^*^	[0.58—1.30]	[0.73—1.50]	[0.63—1.55]	[0.66—1.79]	[1.14—2.15]**	[0.87—2.12]	[0.35—2.17]	[1.42—3.29]^**^	[0.54—2.67]
Asset index	1.31	1.17	0.95	1.06	1.11	1.13	1.19	1.11	1.28	1.05
	[1.19—1.44]^**^	[1.08—1.26]^**^	[0.87—1.04]	[0.98—1.15]	[1.02—1.21]^*^	[1.05—1.22]^**^	[1.12—1.28]^**^	[0.99—1.24]	[1.16—1.42]^**^	[0.88—1.26]
Has any education	0.97	1.44	1.32	1.64	2.39	0.96	1.65	2.29	1.67	2.22
	[0.61—1.53]	[0.90—2.31]	[0.82—2.11]	[0.94—2.85]	[1.42—4.04] ^**^	[0.63—1.48]	[0.95—2.88]	[1.01—5.22]^*^	[1.02—2.74]^*^	[0.84—5.88]
Respondent is female	1.52	0.82	1.31	0.69	2.09	1.44	1.53		1.19	14.23
	[0.93—2.50]	[0.35—1.88]	[0.61—2.84]	[0.31—1.53]	[0.80—5.42]	[0.89—2.34]	[0.60—3.91]		[0.55—2.58]	[0.80—254.52]
Age of Respondent	0.99	1.00	1.00	1.01	1.00	1.00	0.99	0.97	1.00	1.01
	[0.98—1.00]^**^	[0.99—1.01]	[0.99—1.01]	[1.00—1.02]	[0.99—1.01]	[0.99—1.00]	[0.97—1.00]*	[0.94—1.00]*	[0.99—1.01]	[0.99—1.03]
# of children aged 24—35 months	1.75	0.57	0.75	0.61	0.78	1.08	0.61	0.64	1.06	0.36
District (ref: Libo Kemkem)	[1.03—3.00]*	[0.39—0.85]**	[0.47—1.22]	[0.35—1.07]	[0.45—1.37]	[0.72—1.62]	[0.31—1.20]	[0.28—1.46]	[0.54—2.05]	[0.10—1.33]
Ebinat	0.95	0.65	0.84	0.52	0.72	0.92	0.49	1.45	0.32	2.82
	[0.62—1.46]	[0.40—1.06]	[0.59—1.19]	[0.32—0.85]**	[0.42—1.25]	[0.62—1.36]	[0.30—0.80]**	[0.64—3.31]	[0.14—0.72]**	[0.87—9.17]
Dewa Chefa	0.66	0.54	0.82	0.59	0.37	0.84	0.58	0.70	2.05	1.04
	[0.40—1.07]	[0.33—0.88]*	[0.55—1.21]	[0.34—1.03]	[0.22—0.63]**	[0.55—1.30]	[0.32—1.03]	[0.25—1.96]	[1.04—4.03]*	[0.37—2.92]
Artuma Fursi	0.57	0.59	1.18	0.93	0.18	0.66	0.26	0.56	1.14	1.20
	[0.34—0.96]*	[0.36—0.98]*	[0.79—1.77]	[0.55—1.60]	[0.10—0.32]**	[0.42—1.05]	[0.14—0.49]**	[0.22—1.45]	[0.50—2.57]	[0.45—3.19]
*N*	2,493	1,136	1,136	1,134	1,136	2,493	2,493	2,342	2,493	224

*HDA* Health Development Agent, *HP* Health Post, *HEW* Health Extension Worker, *PDS* Permanent Direct Support

The results of the mutually adjusted logistic regression model of health worker and health service access among PW clients are shown in Table 4. Household characteristics positively associated with knowing the community HEW include having children under the age of 5 years in the household, CBHI coverage, household headed by a female, having any versus no education, increased asset index, and any DAS. Having any recent contact with the HEW is positively associated with having children under the age of 5 years in the household and higher asset index. Residents of Dewa Chefa and Artuma Fursi were less likely to have contact with an HEW than residents of Libo Kemkem as were larger households and those with any DAS. Ever being visited by an HEW is positively associated with having any education compared to no formal education. Increased asset index was positively associated with recently being visited by the HEW. Any contact with the HEW outside of the home is positively associated with increased asset index and having any education. Characteristics negatively associated with contact with the HEW outside the home included increasing asset index, any DAS, and residing in Dewa Chefa or Artuma Fursi (versus Libo Kemkem). Having children under the age of 5 in the household, CBHI coverage, any DAS, any education, being female, and increased asset index are positively associated with ever visiting the HP. Living in Artuma Fursi rather than Libo Kemkem was negatively associated with ever visiting a HP. Increased asset index, children under 5 years in the household, CBHI coverage, and having any education were positively associated with recent visits to the HP while residing Artuma Fursi or Dewa Chefa versus Libo Kemkem was negatively associated with recent HP visits. Receiving health advice at the HP was positively associated with having children under 5 years living in the household and increased asset index. Factors positively associated with knowing the HDA include children under 5 years in the household, living in Dewa Chefa relative to Libo Kemkem, any DAS, increased asset index, and having any versus no education. Residing in Ebinat versus Libo Kemkem and increased household size were negatively associated with knowledge of the HDA. Residing in Dewa Chefa versus Libo Kemkem was negatively associated with having contact with the HDA.

**Table 4:** Adjusted odds ratios and 95% confidence intervals for the associations between household characteristics and knowledge of and interaction with health extension workers for PW households (*N* = 2,495)

	**Knows HEW**	**Any contact with HEW (past 3 months)**	**Ever visited by HEW**	**Visited by HEW (past 3 months)**	**Contact with HEW outside home**	**Visited HP**	**Visited HP (past 3 months)**	**Received health advice at HP**	**Knows HDA**	**Had contact with HDA**
Household size	1.00	0.91	0.99	0.96	0.98	0.98	0.94	0.95	0.90	1.03
	[0.94—1.06]	[0.85—0.97]**	[0.92—1.06]	[0.90—1.04]	[0.90—1.06]	[0.92—1.05]	[0.88—1.01]	[0.85—1.06]	[0.82—0.99]*	[0.88—1.20]
Children under 5 in the household	1.86	1.36	1.16	0.98	1.18	2.14	3.40	4.25	1.54	0.54
	[1.52—2.27]**	[1.05—1.76]*	[0.85—1.59]	[0.68—1.42]	[0.86—1.63]	[1.75—2.61]**	[2.37—4.88]**	[2.62—6.87]**	[1.03—2.30]*	[0.26—1.10]
Covered by CBHI	2.28	1.27	1.34	1.31	1.17	1.70	1.69	1.04	1.32	1.16
	[1.82—2.85]**	[0.91—1.75]	[0.99—1.80]	[0.90—1.91]	[0.84—1.65]	[1.35—2.15]**	[1.20—2.40]**	[0.62—1.73]	[0.96—1.82]	[0.56—2.40]
Head is female	1.36	0.86	1.06	1.09	1.03	1.02	1.00	0.83	1.32	1.56
	[1.05—1.76]*	[0.65—1.13]	[0.80—1.40]	[0.79—1.51]	[0.73—1.45]	[0.82—1.27]	[0.73—1.37]	[0.50—1.36]	[0.91—1.92]	[0.85—2.86]
Any distress asset sale (DAS)	1.42	0.70	0.93	0.96	0.63	1.59	1.05	1.00	1.72	1.53
	[1.10—1.84]**	[0.53—0.94]*	[0.69—1.26]	[0.70—1.32]	[0.45—0.87]**	[1.25—2.03]**	[0.71—1.55]	[0.58—1.72]	[1.24—2.40]**	[0.88—2.66]
Asset index	1.26	1.19	1.07	1.13	1.14	1.11	1.25	1.33	1.26	1.12
	[1.15—1.37]**	[1.10—1.30]**	[0.99—1.16]	[1.04—1.23]**	[1.06—1.22]**	[1.05—1.18]**	[1.15—1.35]**	[1.20—1.47]**	[1.17—1.35]**	[0.94—1.33]
Has any education	1.23	1.36	1.76	1.54	1.67	1.69	2.04	1.69	1.51	1.53
	[0.87—1.73]	[0.91—2.02]	[1.20—2.57]**	[0.99—2.39]	[1.09—2.55]*	[1.19—2.41]**	[1.37—3.05]**	[0.97—2.94]	[1.01—2.25]*	[0.71—3.29]
Respondent is female	1.75	1.01	1.84	5.16	1.06	2.32	4.30		1.29	
	[1.01—3.04]*	[0.38—2.71]	[0.62—5.44]	[0.63—42.42]	[0.35—3.19]	[1.17—4.60]*	[0.50—36.97]		[0.43—3.90]	
Age of Respondent	0.98	0.99	1.00	1.00	0.98	1.00	0.99	0.96	0.99	0.99
	[0.98—0.99]**	[0.98—1.00]	[0.99—1.01]	[0.99—1.01]	[0.97—1.00]*	[0.99—1.01]	[0.97—1.00]	[0.94—0.99]**	[0.97—1.00]	[0.96—1.01]
# of children aged 24—35 months	1.29	1.16	1.08	0.87	1.17	1.05	0.94	0.78	0.84	2.27
District (ref: Libo Kemkem)	[0.90—1.85]	[0.82—1.63]	[0.81—1.45]	[0.55—1.38]	[0.78—1.73]	[0.78—1.42]	[0.64—1.38]	[0.47—1.30]	[0.51—1.40]	[0.94—5.45]
Ebinat	0.94	0.71	1.09	0.81	1.06	1.05	0.91	1.74	0.45	1.39
	[0.48—1.82]	[0.45—1.13]	[0.75—1.60]	[0.48—1.35]	[0.64—1.76]	[0.62—1.78]	[0.55—1.52]	[0.85—3.55]	[0.23—0.91]*	[0.46—4.17]
Dewa Chefa	0.66	0.49	0.76	0.82	0.42	0.63	0.52	0.69	1.96	0.43
	[0.33—1.33]	[0.32—0.75]**	[0.54—1.07]	[0.52—1.31]	[0.26—0.69]**	[0.37—1.07]	[0.29—0.93]*	[0.35—1.37]	[1.17—3.30]*	[0.18—0.98]*
Artuma Fursi	0.59	0.64	0.94	1.11	0.28	0.48	0.36	0.65	1.00	0.76
	[0.30—1.17]	[0.44—0.94]*	[0.60—1.49]	[0.68—1.82]	[0.17—0.46]**	[0.29—0.78]**	[0.20—0.65]**	[0.31—1.35]	[0.52—1.94]	[0.38—1.52]
*N*	2,495	1,554	1,554	1,554	1,554	2,495	2,495	2,431	2,495	281

*HDA* Health Development Agent, *HP* Health Post, *HEW* Health Extension Worker, *PW* Public Works

### Characteristics associated with access to social workers and services

The results of the adjusted logistic regression model of access to SWs and social services among PDS clients on various household characteristics are presented in Table 5. Variables positively associated with knowing the SW in the community included any DAS, increased asset index, and having any versus no education. Living in Ebinat, Dewa Chefa, or Artuma Fursi versus Libo Kemkem were all negatively associated with knowing the SW. Having a female household head was positively associated with recent SW contact. Residents of Dewa Chefa were less likely to have recent contact with an SW than those in Libo Kemkem. Residing in Dewa Chefa versus Libo Kemkem was negatively associated with recent visits by the SW and outside contact with the SW. FD attendance was positively associated with having children under 5 years in the household and any DAS. Living in Ebinat (vs. Libo Kemkem) was negatively associated with FD attendance. Recent attendance of a FD was positively associated with having any education.

**Table 5:** Adjusted odds ratios and 95% confidence intervals for the associations between household characteristics and knowledge of and interaction with social workers for PDS households

	**Knows SW**	**Any contact with SW (past 3 months)**	**Ever visited at home by SW**	**Visited by SW (past 3 months)**	**Had contact with SW outside home**	**Attended FD**	**Attended FD (past 3 months)**
Household size	1.03	1.02	0.91	0.78	0.90	1.10	1.19
	[0.92—1.15]	[0.82—1.25]	[0.73—1.12]	[0.57—1.07]	[0.68—1.17]	[0.93—1.30]	[0.87—1.64]
Children under 5 in the household	1.29	0.68	0.43	1.32	1.38	2.36	1.24
	[0.79—2.10]	[0.20—2.34]	[0.13—1.39]	[0.35—5.04]	[0.41—4.66]	[1.08—5.17]*	[0.32—4.84]
Covered by CBHI	1.24	1.74	1.45	1.94	1.53	0.98	1.34
	[0.82—1.89]	[0.88—3.42]	[0.64—3.30]	[0.85—4.46]	[0.54—4.38]	[0.54—1.76]	[0.44—4.10]
Head is female	1.18	3.02	1.49	2.24	1.48	1.66	1.63
	[0.78—1.78]	[1.20—7.57]*	[0.60—3.67]	[0.75—6.74]	[0.58—3.77]	[0.66—4.17]	[0.36—7.39]
Any distress asset sale (DAS)	1.51	1.42	0.93	1.37	0.79	3.35	3.33
	[1.01—2.24]*	[0.63—3.24]	[0.38—2.25]	[0.52—3.63]	[0.23—2.80]	[1.18—9.55]*	[0.42—26.60]
Asset index	1.20	1.06	0.90	0.95	0.96	1.13	1.19
	[1.10—1.31]**	[0.85—1.32]	[0.71—1.14]	[0.73—1.24]	[0.79—1.17]	[0.99—1.30]	[0.98—1.46]
Has any education	2.08	0.40	0.83	0.65	1.03	2.20	6.78
	[1.13—3.82]*	[0.10—1.59]	[0.21—3.36]	[0.11—3.93]	[0.19—5.53]	[0.86—5.60]	[1.79—25.70]**
Respondent is female	0.96	0.77	7.11	2.85	1.27	1.57	
	[0.46—2.01]	[0.13—4.39]	[0.97—52.04]	[0.29—28.06]	[0.13—12.44]	[0.19—12.78]	
Age of Respondent	1.00	0.97	0.99	1.00	0.97	0.98	1.00
	[0.99—1.01]	[0.95—1.00]	[0.97—1.02]	[0.96—1.04]	[0.94—1.01]	[0.97—1.00]	[0.98—1.02]
# of children aged 24—35 months	1.22	0.88	2.46	2.71	0.50	0.57	
District (ref: Libo Kemkem)	[0.55—2.68]	[0.20—3.81]	[0.56—10.77]	[0.55—13.46]	[0.08—3.08]	[0.15—2.11]	
Ebinat	0.19	0.33	0.36	0.28	0.36	0.29	0.47
	[0.10—0.37]**	[0.08—1.46]	[0.12—1.05]	[0.06—1.43]	[0.10—1.27]	[0.11—0.72]**	[0.11—2.00]
Dewa Chefa	0.53	0.25	0.63	0.20	0.25	0.85	0.64
	[0.30—0.93]*	[0.13—0.49]**	[0.29—1.36]	[0.07—0.58]**	[0.11—0.60]**	[0.36—2.02]	[0.11—3.55]
Artuma Fursi	0.17	0.41	0.81	1.10	0.87	0.54	0.73
	[0.09—0.33]**	[0.14—1.24]	[0.27—2.45]	[0.29—4.10]	[0.23—3.27]	[0.14—2.03]	[0.07—7.13]
*N*	2,493	192	192	192	192	2,493	2,238

*SW* Social Worker, *FD* Food Demonstration

Table 6 presents the results of the adjusted logistic regression model for SWs and services access among PW beneficiaries. Factors positively associated with knowing the SW include CBHI coverage, having a female household head, any DAS, increased asset index, and having any versus no education. Residents of Ebinat, Artuma Fursi, and Dewa Chefa relative were all less likely to know a SW than residents of Libo Kemkem. Having any contact with the SW in the past 3 months was negatively associated with increased household size and residence in Artuma Fursi (versus Libo Kemkem). Factors positively associated with FD attendance included increased asset index and having any education. Increased asset index and CBHI coverage were positively associated with recent FD attendance.

**Table 6:** Adjusted odds ratios and 95% confidence intervals for the associations between household characteristics and knowledge of and interaction with social workers for PW households (*N* = 2,495)

	**Knows SW**	**Any contact with SW (past 3 months)**	**Ever visited at home by SW**	**Visited by SW (past 3 months)**	**Had contact with SW outside home**	**Attended FD**	**Attended FD (past 3 months)**
Household size	0.95	0.80	1.09	0.98	0.90	1.02	0.75
	[0.86—1.04]	[0.68—0.94]**	[0.88—1.36]	[0.76—1.26]	[0.78—1.05]	[0.92—1.13]	[0.53—1.05]
Children under 5 in the household	1.20	1.28	0.62	0.55	2.18	1.60	1.45
	[0.82—1.77]	[0.51—3.17]	[0.23—1.69]	[0.18—1.67]	[0.73—6.53]	[0.90—2.84]	[0.60—3.49]
Covered by CBHI	1.53	1.83	0.51	0.64	2.01	1.89	9.45
	[1.07—2.21]*	[0.79—4.23]	[0.25—1.07]	[0.24—1.73]	[0.89—4.56]	[1.09—3.31]*	[1.03—86.38]*
Head is female	1.63	1.72	1.41	1.49	1.93	1.55	0.79
	[1.06—2.50]*	[0.73—4.04]	[0.62—3.20]	[0.70—3.19]	[0.73—5.08]	[0.90—2.68]	[0.10—5.95]
Any distress asset sale (DAS)	1.60	1.79	1.21	1.26	1.23	1.17	1.83
	[1.08—2.37]*	[0.94—3.42]	[0.52—2.83]	[0.55—2.89]	[0.55—2.78]	[0.74—1.86]	[0.56—5.96]
Asset index	1.17	1.16	1.02	1.08	1.14	1.27	1.35
	[1.09—1.25]**	[0.96—1.41]	[0.82—1.28]	[0.86—1.36]	[0.94—1.39]	[1.15—1.40]**	[1.18—1.56]**
Has any education	1.79	1.16	1.32	1.16	0.69	2.55	1.56
	[1.15—2.80]*	[0.44—3.04]	[0.53—3.29]	[0.31—4.30]	[0.23—2.05]	[1.45—4.49]**	[0.78—3.12]
Respondent is female	0.73	0.70	0.62	1.12	0.30		
	[0.25—2.17]	[0.10—5.02]	[0.08—4.95]	[0.08—15.76]	[0.05—2.01]		
Age of Respondent	0.99	1.00	1.01	1.01	1.02	0.98	0.95
	[0.97—1.00]*	[0.96—1.03]	[0.98—1.04]	[0.97—1.04]	[0.99—1.05]	[0.96—1.01]	[0.90—1.00]
# of children aged 24—35 months	0.87	1.62	2.77	2.67	0.93	1.35	1.58
District (ref: Libo Kemkem)	[0.53—1.43]	[0.55—4.79]	[0.79—9.73]	[0.57—12.39]	[0.20—4.34]	[0.74—2.48]	[0.44—5.75]
Ebinat	0.13	0.57	0.47	0.23	0.80	0.90	0.22
	[0.07—0.25]**	[0.21—1.52]	[0.13—1.65]	[0.03—1.68]	[0.31—2.05]	[0.47—1.74]	[0.02—1.99]
Dewa Chefa	0.35	0.52	0.45	0.36	0.74	0.77	0.56
	[0.22—0.56]**	[0.25—1.09]	[0.16—1.29]	[0.10—1.23]	[0.36—1.55]	[0.38—1.56]	[0.11—2.84]
Artuma Fursi	0.18	0.26	0.28	0.44	0.44	0.57	1.34
	[0.10—0.34]**	[0.09—0.73]*	[0.07—1.16]	[0.10—1.88]	[0.13—1.53]	[0.25—1.27]	[0.26—7.01]
*N*	2,495	230	230	230	230	2,431	2,431

*SW* Social Worker, *FD* Food Demonstration

## Discussion

This study descriptively examined knowledge of and interactions with SWs and HEWs among households participating in the PSNP programme. A key finding of this study is that client knowledge of, and interaction with HEWs and SWs is very limited. When SWs interact with PSNP beneficiaries, their wide-ranging support with programme administration, provision of psychosocial services, and case management is generally well received and valued by programme participants. HEWs play an important role in screening pregnant women for TDS and facilitating direct support clients’ access to premium waivers for CBHI. At the same time, inadequate staffing, training, and resources, as well as high staff turnover limit the ability of SWs to effectively carry out their responsibilities.

Our findings are consistent with the existing literature. Higher education of the household head is associated with increased health service utilisation in Ethiopia [24], similar to our results. Similarly, Medhanyie et. al. (2012) also reported that literate women with under-five children are more likely to utilise maternal health services in Ethiopia. Knowledge of SWs and utilisation of their services were associated with increased asset index, having any education, or having a female-headed household in our study. Further, our qualitative findings that access to health facilities is limited by long travel distance, lack of road access or transport services, and lack of ambulances have been shown previously in rural Ethiopia [25, 26].

Our study underscored that PW participants had greater knowledge of HEWs and SWs than PDS clients. Given the greater need for social services due to their inability to work and status as caregivers among PDS participants, this finding highlights a need for better access to information and services among this beneficiary group. Among those who did report knowing the HEW, interactions remained higher among PW than PDS beneficiaries. It is not clear from these data why these unexpected differences are occurring. There are, however, some potential explanations given the characteristics of the sample and the programme implementation. First, the SW cadre was not yet completely established in all areas of Dewa Chefa, whereas the SW in Libo Kemkem had been operating for two years prior to this baseline data collection [16]. Second, the same characteristics that may contribute to PDS eligibility (having no able-bodied workers in the household, often being elderly, people with disabilities and chronic illness, and orphaned children with no support) may also result in stymied frontline agent interactions as the beneficiaries may not be able to leave their homes and traverse the difficult terrain or afford transportation to see HEWs at health posts. The transportation constraint operates in both ways, meaning that HEWs and SWs may not have the resources or time to visit homes that are great distances from the communities where they primarily work [15]. PW clients are also more likely to attend BCC activities on maternal and child nutrition which are facilitated by HEWs compared to members from PDS households. This increases opportunity for PW households to interact and gain access to HEWs.

This study builds on an existing literature on integrated social protection programming and outcomes that has identified both supply- and demand-side barriers to improved access to frontline agents and use of their services. In support of the qualitative findings regarding barriers to frontline worker access, the ability to adequately carry out job roles and responsibilities is often undermined by low salaries, insecure job contracts and overwhelming workloads that result in worker attrition and high staff turnover [27]. The perpetual cycle of high staff turnover resulting from low pay and encumbering workloads heavily affected the work forces as many were unqualified for their work in these communities or did not have the support and/or resources to carry out their assigned tasks, as alluded to in our qualitative interviews and found in previous studies [7, 8, 28]. These issues were most relevant, but not exclusive, to SWs in our study. This is a salient point to consider as, in the context of the ISNP, SWs are pivotal in facilitating linkages to available social services among beneficiaries and other frontline workers while overseeing case management for marginalised PDS households [29]. Thus, inadequate work or knowledge on the part of the SWs could significantly affect the implementation of integrated programming as designed.

Additionally, frontline agents are subject to logistical and budgetary constraints that impair their ability to communicate with (via phone) or visit clients as transportation to the widely dispersed households given the road conditions of the areas have been shown to be difficult [15]. ISNP beneficiaries reported having their own specific limitations to participating in the activities outlined by the programme in qualitative interviews. To begin with, knowledge of frontline workers was low in the population examined, particularly for SWs. Similar findings were presented in the IN-SCT pilot evaluation, conducted among a similar population of PSNP households, in that participant knowledge of SWs in particular was low [7]. Further, financial constraints restrict participants’ abilities to use services, either due to costs around travel or for services purchased (for example, health care services) [12].

Given the critical role served in the programme by SWs, it is important to understand the limitations in knowledge of SWs and interaction to improve their relationships and the outcomes of interest among these populations in which SWs work. It is also not entirely clear why knowledge of SWs is so low with less than 5% of either PW or PDS beneficiaries having any contact with, being visited by, or having a recent visit by the SW. Limited knowledge of and interactions with SWs may reflect the myriad roles and responsibilities attributed to their jobs including monitoring and data collection if households comply with co-responsibilities such as sending children to school and pregnant mothers are exempt from public works and the lack of priority of client interactions [7, 8, 28]. SWs also engage in targeting process as a member of the kebele (community) food security task force (MoA, 2014) which further increases their work responsibilities. It is notable, however, that those who do know and interact with SW consider them to serve an indispensable role in improving livelihoods and the community as a whole [27]. The prevailing logistic or technical constraints may also hinder SW-client interactions, so more support and resources should be lent to SWs in to appropriately implement ISNP and facilitate these linkages to improve outcomes among these communities. Given the early stage of the pilot at the time of this study data collection and that ISNP had not yet been implemented, these outcomes may improve as features of the programme are introduced, and these agents are completely mobilised.

There are some limitations to the study. The cross-sectional nature of this study prohibits us from concluding that the relationships examined are causal. The study sample is not representative of the broader population of Ethiopia as those targeted in this sample were living in extreme poverty in rural regions of the country. However, the large, random sample enables us to generalise to the PSNP population. The extent of data collected from these household interviews and KIIs with important agents in the PSNP/ISNP scheme gathered comprehensive information that enables exploration of multiple linkages and prevailing themes influencing frontline agent-client connections.

## Conclusions

Frontline workers are a key cadre in implementing intersectoral linkages and case management, but are often understaffed, poorly trained, and under-resourced. Studies of the PSNP have shown that the programme improves household-level food security but often fails to deliver on nutritional status and anthropometric outcomes, indicating a critical need for more intersectoral, integrated programming targeted to participants of the PSNP, which can only be achieved through coordination efforts of frontline workers. Our findings underscore gaps in both the supply- and demand-side barriers to frontline worker access and utilization of services which must be addressed before the full potential of integrated programming can be achieved.

## Supplementary Information

**Additional file 1: Supplementary Figure 1.**

**Additional file 2:** **Supplementary Table 1. **Study design and sampling information

**Additional file 3:** **Supplementary Table 2. **Robustness check results

## Data Availability

Data are not currently publicly available and cannot be made available upon request due to Government processes (the Government of Ethiopia and UNICEF jointly own the data). Data are expected to be made publicly available, subject to approval by the Government of Ethiopia and UNICEF, no sooner than one year after the publication of the final impact evaluation report.

## Abbreviations

BCC: Behaviour Change Communication
BoLSA: Bureau of Labor and Social Affairs
BoWCA: Bureau of Women and Children Affairs
CBHI: Community-based health insurance
CCC: Community Care Coalition
DA: Development Agent
DAS: Distressed asset sale
ECO: UNICEF Ethiopia Country Office
EDHS: Ethiopia Demographic and Health Survey
FD: Food demonstrations
FGDs: Focus group discussions
GoE: Government of Ethiopia
HEW: Health Extension Worker
HP: Health post
IDIs: In-depth interviews
IN-SCT: Integrated Basic Social Services and Social Cash Transfer
ISNP: Integrated Safety Net Programme
KIIs: Key informant interviews
MIS: Management Information System
MoLSA: Ministry of Labor and Social Affairs
NSPP: National Social Protection Policy
NSPS: National Social Protection Strategy
OoR: UNICEF Office of Research
PDS: Permanent Direct Support
PSNP: Productive Safety Net Programme
PW: Public Works
SCTPP: Tigray Social Cash Transfer Pilot programme
SDGs: Sustainable Development Goals
SNNP: Southern Nations, Nationalities, and People’s
SPAP: Social Protection Action Plan
SW: Social Worker
TDS: Temporary Direct Support
WFS: Woreda Food Security
WoLSA: Woreda Office of Labour and Social Affairs
WoLSA: Woreda Office for Labor and Social Affairs
