# Supplementary figures and images for: Knowledge of and access to frontline workers among poor, rural households in Amhara region, Ethiopia: a mixed-methods study
Source: BMC Public Health. 2022 Nov 25;22:2179. doi: 10.1186/s12889-022-14594-8 (PMC9700966; doi:10.1186/s12889-022-14594-8)

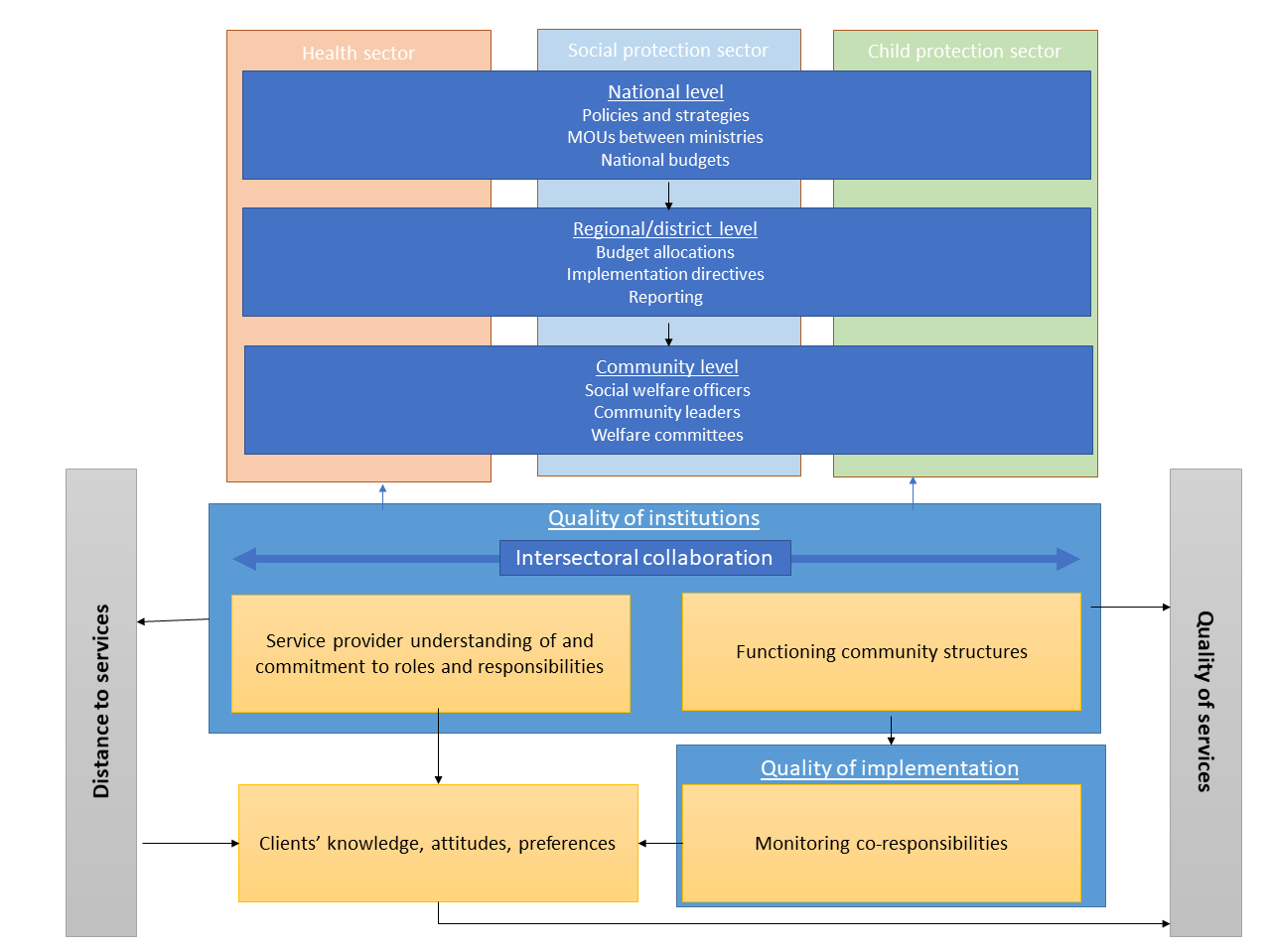

Supplement: Supplementary file 1 — Additional file 1: Supplementary Figure 1. [file 12889_2022_14594_MOESM1_ESM.png]
