# Supplementary material for: Knowledge of and access to frontline workers among poor, rural households in Amhara region, Ethiopia: a mixed-methods study
Source: BMC Public Health. 2022 Nov 25;22:2179. doi: 10.1186/s12889-022-14594-8 (PMC9700966; doi:10.1186/s12889-022-14594-8)
Supplement: Supplementary file 2 — Additional file 2: Supplementary Table 1. Study design and sampling information [file 12889_2022_14594_MOESM2_ESM.docx]

**Supplementary Table 1. Study Design and Sampling Information**

Two are treatment woredas (Libo Kemkem and Dewa Chefa) targeted for the pilot and two are comparison woredas (Ebinat and Artuma Fursi). These woredas were purposively selected through an extensive process which identified the treatment woredas based on an inclusive approach with stakeholders as well as on objective needs/capacity-based assessment. Needs were assessed in the areas of education, nutrition, and health. Additionally, woredas were assessed in the areas of health insurance (availability of CBHI at woreda level), nutrition (nutrition interventions in place), linkage and coordination between Woreda Office of Labour and Social Affairs (WoLSA) and Woreda Food Security (WFS)/Nutrition sensitive agriculture, managerial, technical and administrative capacity, linkages to other UNICEF interventions, and accessibility and practicality for UNICEF support. Study populations were selected based on different characteristics related to household composition and income-generating capacity of the household. Comparison woredas were selected by knowledgeable staff of the UNICEF Country Office (ECO) who had a thorough understanding of the characteristics of these woredas. These comparison woredas (Ebinat and Artuma Fursi) were selected based on characteristics similar to treatment areas such as socio-demographic profile, health service supply, program
