# Supplementary material for: Knowledge of and access to frontline workers among poor, rural households in Amhara region, Ethiopia: a mixed-methods study
Source: BMC Public Health. 2022 Nov 25;22:2179. doi: 10.1186/s12889-022-14594-8 (PMC9700966; doi:10.1186/s12889-022-14594-8)
Supplement: Supplementary file 3 — Additional file 3: Supplementary Table 2. Robustness check results [file 12889_2022_14594_MOESM3_ESM.docx]

**Supplementary Table 2. Robustness Check Results**

Results of the robustness checks suggest that inclusion and control of the age variable in our analyses is warranted. The mean sample characteristics for both PW and PDS clients in the main sample were compared to those of the full sample and showed that most of the differences in characteristics’ averages were small (less than 1 percentage point difference). However, the average composition of females in the main sample were shown to be 6 and 5 percentage points higher than the full sample for PW and PDS clients, respectively. For the sample outcomes robustness test, there were no observed differences in means for the PW clients. The differences observed between the PDS clients of the main and full samples were small (less than 1 percentage point). Differences in significance observed for the results of the health worker PDS regressions are most common for education and female respondent variables. Other variables that have significance in one sample regression while absent in the other are household size, asset index, and residing in Dewa Chefa (versus Libo Kemkem). In the health worker PW regression analysis, the odds ratios differ most with respect to household size and education and to a lesser extent female respondent and residence in Dewa Chefa relative to Libo Kemkem. In comparison to the full sample, the regression results for social worker access among PW clients differ most for female household head, education, and asset index. The variables that differ in significance between the two samples in the social worker regression analysis for PDS clients include female household head, asset index, education, and female respondent.
